# The Management of Horses during Fireworks in New Zealand

**DOI:** 10.3390/ani6030020

**Published:** 2016-03-09

**Authors:** Gabriella Gronqvist, Chris Rogers, Erica Gee

**Affiliations:** Massey Equine, Institute of Veterinary, Animal and Biomedical Sciences, Massey University, Private Bag 11-222, Palmerston North 4442, New Zealand; c.w.rogers@massey.ac.nz (C.R.); e.k.gee@massey.ac.nz (E.G.)

**Keywords:** fireworks, horses, anxiety, behaviour, fear

## Abstract

**Simple Summary:**

The negative effects of fireworks on companion animals have been reported, but little has been documented on the impact on horses. Horse anxiety was commonly associated with fireworks, and 26% of owners reported horse injuries as a result of fireworks. Many management strategies were seen as ineffective. The majority of horse owners were in favour of a ban on the sale of fireworks for private use.

**Abstract:**

Within popular press there has been much coverage of the negative effects associated with firework and horses. The effect of fireworks has been documented in companion animals, yet no studies have investigated the negative effects, or otherwise, of fireworks on horses. This study aims to document horse responses and current management strategies to fireworks via an online survey. Of the total number of horses, 39% (1987/4765) were rated as “anxious”, 40% (1816/4765) “very anxious” and only 21% (965/4765) rated as “not anxious” around fireworks. Running (82%, 912/1107) was the most common behaviour reported, with no difference between property type (*p* > 0.05) or location (*p* > 0.05). Possibly as a consequence of the high frequency of running, 35% (384/1107) of respondents reported having horses break through fences in response to fireworks and a quarter (26%, 289/1099) reported that their horse(s) had received injuries associated with fireworks. The most common management strategy was moving their horse(s) to a paddock away from the fireworks (77%) and to stable/yard them (55%). However, approximately 30% reported these management strategies to be ineffective. Of the survey participants, 90% (996/1104) were against the sale of fireworks for private use.

## 1. Introduction

In New Zealand, both public and private firework displays are common, especially during Guy Fawkes Day on 5 November. The Hazardous Substances and New Organisms Act 1996 govern the sale of fireworks for private use and limits sales to three days (2–5 November). However, while the sale of fireworks is restricted to only three days, there are no restrictions on the when fireworks can be used. Many counties, such as Canada, South Africa, Australia and Finland, have strict limitations or bans on private firework displays. A recent Parliamentary select committee rejected a ban on the private use of fireworks in New Zealand citing any changes as unnecessary and unenforceable [1]. The lack of change to the Hazardous Substances and New Organisms Act 1996 relating to the use of fireworks has prompted initiation of a subsequent petition calling for the ban of private use of fireworks [2]. The petition has the support of the New Zealand Police, Fire services and New Zealand Society for the Prevention of Cruelty to Animals (NZSPCA).

Previous studies have reported on fear behaviours in response to fireworks in companion animals both in New Zealand [3] and internationally [4]. Owners have reported negative effects of fireworks including escaping, vocalisation, urination or defecation, trembling and destructive behaviour.

Within the popular press and social media there has been much coverage of the negative effects associated with firework displays and horses [5]. Nevertheless, no studies appear to have investigated the possible problems, or otherwise, of fireworks on horses. Despite this lack of reporting in the peer reviewed literature, there are a number of publications from equestrian organisations and within the equestrian press providing guidelines on the management of horses and the negative effects of fireworks displays [6].

This lack of scientific literature on the topic may relate to the intensive management of horses within stables and therefore reduction in the exposure to the potentially noxious stimuli of fireworks. In November, in the United Kingdom approximately 70% of horses are stabled and 30% live outside. Of those horses that are stabled, almost 50% spend between 9 and 16 h inside daily [7]. The management of horses, even high level competition horses, in New Zealand is unique in that the temperate climate permits management of the horse at pasture year round [8]. This pastoral based management system may facilitate exposure to the visual, acoustic and olfactory stimuli of fireworks and be a reason for the perception that the private use of fireworks represents a hazard for horses at pasture.

In animals, fear responses to fireworks are believed to occur due to the intermittent and unpredictable high-intensity noise [9]. Cracknell and Mills (2008) report that the effects of secondary stimuli such as odours, light flashes and changes in barometric pressure on animals still remain largely unknown.

Horses are generally considered to be highly unpredictable flight animals [10] shown to be reactive to loud noises and flashing lights [11]. Fear is a reaction to perceived danger and is characterized by physiological and behavioural changes that heighten the individual’s ability to deal with that danger [12,13]. Fear based behaviours in horses are numerous and include running, sweating and trembling [14]. Flight responses are particularly dangerous, with the potential to result in severe accidents of the horse and rider/handler [15].

At present, the lack of data on management strategies employed by horse owners, the perceived effectiveness of such changes and injuries encountered limits debate on the private use of fireworks and the consequences to horses. The aim of this study was to document horse responses and current management strategies to fireworks via an online survey.

## 2. Experimental Section

### 2.1. Questionnaire

Data were collected via an online survey using commercial survey software SurveyMonkey Audience (SurveyMonkey Inc.) (see supplementary file). The survey was initially distributed and “seeded” via six national and regional equestrian sport social media sites. The survey was open for 19 days from 14 October to 1 November 2015, prior to the first official day of the sale of fireworks for private use (2 November) in New Zealand.

The questionnaire could only be completed once per computer and all applicants remained anonymous. The survey was deemed to be low risk by the Massey University Human ethic committee and was registered as a low risk notification project.

The questionnaire consisted of 15 multi-choice and open ended questions in four categories covering property location and size, number of horses and primary use, the reaction of horses to fireworks in the previous year, preventative management and the occurrence of any injuries. Lastly, the participants were asked whether they were in favour of sale of firework for personal use.

### 2.2. Statistical Analysis

Data were described using simple descriptive statistics. In some instances the respondent may not have completed all questions and so the denominator for some questions may vary. For the data on anxiety and horses, the percentages reported here were based on the number of horses for which an anxiety score was given by the respondents. The distribution of property type, horse ownership, behaviour of horses during firework displays and owner support of the sale of fireworks for personal use were examined using a Chi squared test. The differences in behaviours reported between property types were tested using the Mann-Whitney test. Multivariate logistic regression was used to calculate the Odds Ratio (95% confidence intervals) of horse injury with property type and area of where the horse was kept. All statistical analyses were completed using the statistical software STATA 12 (StataCorp, TX, USA) and R 3.2.2 (Foundations for Statistical Computing, Vienna, Austria) with *p* < 0.05 set for significance.

## 3. Results

### 3.1. Demographics

Data were collected from 1111 respondents responsible for 6431 horses. It is estimated there are 110,000 horses in New Zealand and the AgriBase database identifies 13,072 properties with horses not identified as racing or commercial breeding properties [16]. Using these 13,072 properties as an approximate sampling frame, this represents a return rate of 9% (1111/13,072) of (non-racing) horse owners/properties in New Zealand. The majority of the respondents were from the North Island (89% 918/1111) and the greatest number of respondents were from the Auckland region (27%, 295/1111), followed by the Manawatu-Wanganui region (14%, 150/1111, Table 1).

Approximately half the respondents identified themselves as living in an area consisting of predominantly lifestyle blocks (small farms < 10 ha, Table 2). Irrespective of the urban/rural classification, most respondents identified they lived and kept horses on lifestyle blocks (71%, 807/1107) with only 13% (130/1107) of respondents keeping horses on agistment properties (livery service). There were greater numbers of horses kept on an agistment property (15 (interquartile range IQR 7–26)) than on farms (commercial) or lifestyle blocks (5 IQR 3–10 *vs.* 4 IQR 2–6, *p* < 0.001). The number of horses owned by respondents also differed across property type, farm 4 (IQR 3–9), Lifestyle 4 (IQR 2–6), and agistment 4 (IQR 1–12), (*p* < 0.001). The most common use of horses were for sport (show jumping, dressage and eventing) (43%, 1979/4575) followed by trekking (25%, 1137/4573) Pony Club (17%, 774/4575) and racing (7%, 321/4575). The remaining categories (hunting, endurance and western) accounted for approximately 3% each.

Of the total number of horses, 39% (1987/4765) were rated as “anxious”, 40% (1816/4765) “very anxious” and only 21% (965/4765) were rated as “not anxious” around fireworks or the Guy Fawkes period (Table 2). The levels of anxiety reported did not differ between property type (agistment, farm or lifestyle block, *p* > 0.05).

### 3.2. Adverse Horse Behaviour and Anxiety

**Figure 1:**
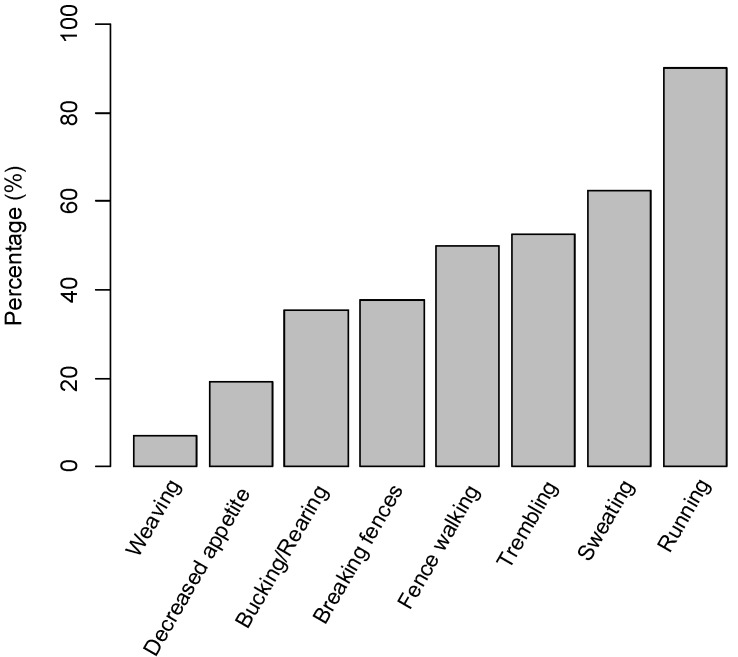
The percentage (%) of participants reporting that their horse(s) had exhibited the behaviors in association with fireworks.

The majority of respondents (1104/1111) reported that their horse(s) had previously exhibited at least one of the behaviours, listed in Figure 1, in response to fireworks. Running (82%, 912/1107) was the most common behaviour reported, with no significant difference between property type (*p* = 0.412) or location (*p* = 0.068). There were a group of behaviours with similar frequencies reported (trembling, sweating and fence walking). Similar frequencies of these behaviours were reported across property location identifiers (urban, semi-rural, lifestyle or rural). Possibly as consequence of the high frequency of running, 35% (384/1107) of respondents reported having horse(s) break through fences in response to fireworks.

A quarter (26%, 289/1099) of participants reported that their horse(s) had received injuries associated with fireworks. Multiple different injuries were reported; the most common were lacerations (40%, 194/289), strains/sprains (10%, 33/289) and broken limbs (7%, 11/289). The property type did not affect the odds of the horse(s) receiving injuries (*p* > 0.05, agistment = reference level, farm 1.5 (0.85–2.48), lifestyle 1.1 (0.71–1.7)). However, respondents that kept horses in rural areas were 0.6 (0.33–0.49) times as likely to report that their horses had received injuries due to fireworks, than respondents that kept horses in an area surrounded by lifestyle blocks (*p* < 0.05, reference level). The odds of horses receiving injuries did not differ between horses kept in semi-rural areas or urban areas compared with lifestyle blocks.

### 3.3. Duration of Firework Displays

Off the survey participants, 6% (63/1108) reported that their horse(s) had not been exposed to fireworks in the previous year. The remaining 94% (1045/1108) of survey respondents were asked the duration of time their horse(s) were exposed to intermittent fireworks. Thirty-three per cent (371/1108) reported fireworks continued for one or two weeks after Guy Fawkes, 26% (288/1108) for two or more months after Guy Fawkes, and 19% (209/1108) for up to a month after Guy Fawkes, while only 16% (177/1108) identified that their horse were exposed to fireworks only on Guy Fawkes Day.

### 3.4. Owner Management Strategies

The most common management strategy was the movement of the horse(s) to a paddock away from the fireworks (77% (779/1006)). However, 37% (374/779) reported this management strategy to be ineffective in reducing anxiety. Horse(s) had previously been stabled or yarded during fireworks by 55% (461/925) of respondents, but 30% (277/461) reported this to be ineffective. Only 30% (254/845) and 19% (152/808) of participants, respectively, had previously either moved their horse(s) off the property or sedated their horse(s) during fireworks. In both instances, 9% (73/254 and 66/152, respectively) of respondents deemed these approaches to be ineffective.

When asked about future management strategies, 20% (189/987) reported that they had no strategy planned. Of the participants, 55% (570/987) planned to move their horse(s) to a paddock further away from the fireworks, 24% (241/987) planned to stable or yard their horse(s), 12% (114/987) planned to sedate them and 10% (95/987) move them off the property. Participants were able to report on several management strategies.

### 3.5. The Sale of Fireworks for Private Use

Of the survey participants 90% (996/1104) reported that they did not support the sale of fireworks for private use, while 10% (108/1104) supported it. The majority of participants did not support the sale of fireworks even if their horse(s) had not previously been injured due to fireworks (χ^2^ = 17.917, df = 1, *p* < 0.05). A greater proportion of participants that kept their horses on lifestyle blocks did not support the sale of fireworks for private use (χ^2^ = 16.799, df = 2, *p* < 0.05), compared to those who kept horses on farms or agistment.

## 4. Discussion

The distribution of responses to the online survey was similar to that obtained by Rosanowski, Cogger, Rogers, Benschop and Stevenson [16] using a generalised random-tesselation stratified sampling design and indicates good agreement and reflection of the distribution of horse ownership location within New Zealand. The overrepresentation of the North Island may reflect that the surveys were seeded initially from social media sources based in the Manawatu and the North Island of New Zealand. The high level of response from respondents on lifestyle blocks was reflected in the bulk of respondents keeping their horses on their own property, rather than using agistment/livery yards, as is an option in Western Europe. Lifestyle blocks are typically less than 4 ha and so within a geographical proximity to neighbours where fireworks displays would provide obvious visual and auditory stimuli. The majority of the respondents kept horses for sport, rather than racing, which reflects the initial sampling frame of the survey and the pattern of horse ownership previously reported in New Zealand [16].

The timing of the survey was intentional to provide an overview of what was planned as a course of action during the “fireworks season”. Sampling at this time provided minimisation of temporal bias, which is often a limitation in survey data the greater the duration between the event and the collection of the data. The survey was closed prior to the first official sale of fireworks and thus avoided bias in responses, or type of respondent, if adverse fireworks events were reported within the press. Motivation to complete the survey may have been greater in participants that had previously experienced an adverse event associated with fireworks. However, the large number of respondents should have attenuated this bias and implies that, within a pastoral management system, negative experiences with horses and fireworks are the norm rather than an exception. The inability to provide a tight definition around the term anxious and very anxious means some caution should be used when differentiating between these behaviour categories. Within the literature, fear and an ethogram for fear is well described. The use of a grading scale for stress could have been used to provide a tighter definition of the level of anxiety (stress) the horse experienced during fireworks displays [17]. However, in an attempt to increase opportunity for initiation and completion of the survey, the complexity of the anxiety was kept to simple low resolution descriptors. The objective of the survey was to obtain data on owners’ perceptions, and general management strategies of their horses in relation to fireworks and not precisely quantify the level of anxiety/arousal to fireworks. Within the literature there are data on between breed and between individual levels of responsiveness to stimuli. These can also be tempered by changes in management. This is an area of behaviour research that requires investigation and possibly translation/dissemination to provide pragmatic management strategies for New Zealand horse owners during fireworks season.

Almost 80% of survey participants reported that their horses became anxious or very anxious during firework displays, with the remaining rating their horses as not anxious. These results support research by Young, Creighton, Smith and Hosie [17] who reported that the sound of fireworks played from compact disk caused higher cortisol levels in horses than the sound of coat clippers or social isolation. The present results are also in agreement with the fact that the majority of respondents described their horse(s) as presenting with, at least one, anxiety related behaviour during fireworks. The most common observation of “running” reflects the use of the flight response to escape noxious stimuli and the pasture based management system of horses in New Zealand. These factors are also reflected in the fact that almost 40% of respondents reported that their horse(s) had broken through fences.

A quarter of respondents reported that their horse(s) had received injuries they believed to be a result of the firework display. The injuries ranged from minor cuts and sprains to broken limbs resulting in death. The most prevalent injuries reported were lacerations varying from mild to severe. These injuries are possibly a reflection of the high percentage of horses reported to break through fences.

Respondents were asked whether, in previous years, they had moved their horse(s) to a paddock away from the fireworks displays, stable/yarded their horse(s), moved their horse(s) away from the property or sedated their horses during Guy Fawkes Day. Most owners had previously trialled a number of management strategies. The most prevalent management approaches were moving the horse(s) to a paddock further away from the fireworks or to stable/yard them. However, almost 40% deemed these methods unsuccessful in reducing anxiety. Nevertheless, these were also reported to be the two most common future management strategies, possibly as they are easier alternatives to relocating the horse off the property or sedation. It has previously been reported that the most common management methods of companion animals owners during fireworks are keeping the animals inside, comforting them, keeping blinds shut and distracting them with music [3]. The majority of these distraction strategies are not likely to be suitable for horses, especially in New Zealand where pastoral management systems are the norm. Moreover, trying to comfort or move a panicked horse can be dangerous for both the handler and the horse as they can charge blindly into humans, fences or other structures when highly aroused [15].

Habituation to repeatable stimuli often occurs with horses, as long as the behaviour is not reinforced with an adverse event [18,19]. The keeping of horses at pasture should permit exposure to fireworks and the opportunity for habituation. This may not, however, be occurring due to the generally focused exposure around the date of Guy Fawkes (November 5th) and then often intermittent exposure. A third of participants reported that they were exposed to fireworks for one or two weeks after Guy Fawkes. Another quarter of participants reported on exposure of two or more months after Guy Fawkes. However, the intermittent and possibly short bouts of exposure may not be enough to habituate horses. Furthermore, the lack of ability to plan and manage horses safely around fireworks has been cited repeatedly in social media and popular press articles [20].

The majority of participants reported that they did not support the sale of fireworks for personal use. This reflects the large number of participants who reported that their horse(s) have displayed anxiety and anxiety related behaviours during fireworks. It is possible that horse owners who have previously had negative experiences, such as injuries, were more motivated to respond to this survey. However, when asked whether the participants supported the sale of fireworks for private use, the majority answered “no”, regardless of whether their horse(s) had previously been injured due to fireworks or not. Moreover, the large sampling size should have damped a potential bias. A greater proportion of participants whose horses were kept at lifestyle blocks (as compared to farms and agistment) were against the sale of fireworks for personal use. This may be a reflection of the relative close proximity to neighbouring properties and perhaps a greater number of neighbouring properties.

## 5. Conclusions

This study is the first to address the issue of horses and fireworks in New Zealand. The article provides a framework for discussing and reviewing legislation in relation to firework use and the risks posed or perceived by horse owners during Guy Fawkes Day firework displays.

## Figures and Tables

**Table 1:** The total number and percentage (%) of respondents from the various regions in New Zealand and the number of property type (agistment ^1^, farm ^2^ or lifestyle ^3^) in the various regions.

Area	N Respondents	% NZ	N Agistment	N Farm	N Lifestyle
Auckland	295	26.6	55	27	213
Bay of Plenty	45	4.1	2	2	41
Canterbury	91	8.2	11	12	68
Gisborne	7	0.6	-	-	7
Hawke’s Bay	38	3.4	6	3	29
Manawatu	150	13.5	15	32	102
Marlborough	17	1.5	-	3	14
Nelson	3	0.3	1	-	2
Northland	61	5.5	3	11	46
Otago	56	5.1	2	20	34
Southland	13	1.2	-	3	10
Taranaki	72	6.5	8	12	52
Tasman	4	0.4	-	-	4
Waikato	125	11.3	12	22	91
Wellington	125	11.3	14	20	89
West Coast	8	0.7	1	2	5

^1^ Livery service; ^2^ Commercial farming enterprise; ^3^ Small farm <4 ha in total area.

**Table 2:** The total number of respondents and horses according to location, property type and the behaviours exhibited during fireworks.

Categories	Total	Urban^1^		Semi-Rural^2^		Lifestyle^3^		Rural^4^	
	n	n	%	n	%	n	%	n	%
Respondents	1111	80	7.2	271	24.4	517	46.5	242	21.8
Property type									
Farm		7	8.8	34	12.5	23	9.5	106	20.5
Lifestyle block		28	35.0	193	15.9	457	188.8	112	21.7
Agistment		45	56.3	43	71.2	36	14.9	23	4.4
Horses/respondents (median and IQR)		3 (2–8)		4 (2–7)		4 (2–5)		4 (2–7)	
**Behaviours**									
Fence walking		39	48.8	136	50.2	237	45.8	94	38.8
Running		67	83.8	223	82.3	444	85.9	179	74.0
Decreased appetite		17	21.3	62	22.9	86	16.6	30	12.4
Breaking through fences		37	46.3	108	39.9	170	32.9	69	28.5
Weaving		9	11.3	19	7.0	14	2.7	34	14.0
Bucking/rearing		29	36.3	100	36.9	157	30.4	72	29.8
Sweating		46	57.5	156	57.6	316	61.1	114	47.1
Trembling		39	48.8	137	50.6	266	51.5	92	38.0
**Injuries**									
YES		25	33.3	84	33.3	138	28.9	42	20.4
Anxiety (n horses)	4765	338		1226		2113		1088	
not anxious		44	13.0	219	17.9	382	18.1	320	29.4
anxious		166	49.1	513	41.8	892	42.2	355	32.6
very anxious		128	37.9	494	40.3	839	39.7	413	38.0
Against the sale of fireworks for personal use		78	97.5	243	89.7	473	91.5	202	83.5

^1^ within a town/urban environment; ^2^ adjacent to an urban area; ^3^ surrounded by lifestyle blocks; ^4^ surrounded by other large farms.

## Supplementary Materials

The following are available online at www.mdpi.com/2076-2615/6/3/20/, Questionnaire: Owner management of horses during Guy Fawke's Day.
