# Supplementary material for: The Management of Horses during Fireworks in New Zealand
Source: Animals (Basel). 2016 Mar 9;6(3):20. doi: 10.3390/ani6030020 (PMC4810048; doi:10.3390/ani6030020)
Supplement: Supplementary file 1 [file animals-06-00020-s001.pdf]

Supplementary Materials:

The Management of Horses during Fireworks in New Zealand

Gabriella Gronqvist *, Chris Rogers, and Erica Gee

Owner management of horses during Guy Fawkes Day.

This study aims to examine how horses are managed during Guy Fawkes day in New Zealand and horse owners' perception of private firework displays. Any information you provide is anonymous and not linked back to the respondents.

MASSEY UNIVERSITY
TE KUNENGA KI PŪREHUROA
UNIVERSITY OF NEW ZEALAND

THE ENGINE OF THE NEW NEW ZEALAND

Owner management of horses during Guy Fawkes Day.

1. **Please tick your geographic area:**

- ◯ Northland
- ◯ Auckland
- ◯ Waikato
- ◯ Bay of Plenty
- ◯ Gisborne
- ◯ Hawke's Bay
- ◯ Taranaki
- ◯ Manawatu-Wanganui
- ◯ Wellington
- ◯ Tasman
- ◯ Nelson
- ◯ Marlborough
- ◯ West Coast
- ◯ Canterbury
- ◯ Otago
- ◯ Southland

2. **What is the best definition of the location of the property where your horse(s) are kept?**

- ◯ Rural (surrounded by other large farms)
- ◯ Lifestyle (in an area immediately surrounded by lifestyle blocks)
- ◯ Semi-rural (adjacent to an urban area)
- ◯ Urban (within a town/urban environment)

Other (please specify)

3. **What is the best definition of the property type where your horse(s) are kept?**

- ◯ Farm (commercial)
- ◯ Lifestyle block (< 10ha)
- ◯ Equestrian centre

Other (splease specify)

4. Do you live on this property?

- ◯ Yes
- ◯ No

5. What is the total number of horses kept on this property?

6. **Please indicate the number of horses you keep on this property and their main purpose:**

Sport (SJ / Dr / Eventing)

Pony Club

Hunting

Endurance

Western

Trekking / Pleasure

Racing – Thoroughbred

Racing – Standardbred

Breeding

Other

7. **How anxious would you rate your horse(s) during firework displays?**

	Not anxious	Anxious	Very Anxious
Horse 1	○	○	○
Horse 2	○	○	○
Horse 3	○	○	○
Horse 4	○	○	○
Horse 5	○	○	○
Horse 6	○	○	○
Horse 7	○	○	○
Horse 8	○	○	○
Horse 9	○	○	○
Horse 10	○	○	○

8. **Which of the following behaviours have your horse(s) exhibited during firework displays?**

- ◯ Fence walking
- ◯ Running
- ◯ Decreased appetite
- ◯ Breaking through fences
- ◯ Weaving
- ◯ Bucking / rearing
- ◯ Sweating
- ◯ Trembling

Other (please specify)

9. **How long did these behaviours last?**

- ◯ N/A
- ◯ The duration of the firework display
- ◯ Up to 2 hours after the firework display ended
- ◯ Until the next day

10. **Did the horse(s) injure themselves as a result of fear/anxiety caused by the firework display?**

- ◯ N/A
- ◯ Yes
- ◯ No

11. Please describe the horse's injury/injuries and if the injury/injuries were mild, moderate or severe

12. **Please indicate if you have tried the following management strategies for your horse(s) during firework displays in previous years and how effective the management strategy was.**

	Not effective	Effective	Very effective	N/A
Move horse(s) to a paddock away from firework displays	◯	◯	◯	◯
Stable / yard horse(s)	◯	◯	◯	◯
Move horse(s) off property	◯	◯	◯	◯
Sedate horse(s)	◯	◯	◯	◯

Other (please specify)

13. **Last year in your area, which is the best descriptor of when your neighbours last used fireworks?**

◯ Never

◯ Only on Guy Fawkes Day

◯ For one to two weeks after Guy Fawkes Day

◯ For up to a month after Guy Fawkes Day

◯ For two or more months after Guy Fawkes Day

14. **How do you plan to manage your horse(s) this coming Guy Fawkes's Day?**

◯ No management strategy

◯ Move horse(s) to a paddock away from fireworks displays

◯ Stable/yard horse(s)

◯ Move horse(s) off property

◯ Sedate horse(s) (e.g. dormosedan, ACP, herbal calmer, etc.)

Other (please specify)

15. **Are you in favour of sale of fireworks for personal use?**

◯ Yes

◯ No
